# Causal effect of life-course adiposity on the risk of respiratory diseases: a Mendelian randomization study

**DOI:** 10.1186/s12986-025-00915-2

**Published:** 2025-03-21

**Authors:** Xi Xi Chen, Fang Ying Lu, Yi Wang, Liu Zhang, Shi Qi Li, Ying Ni Lin, Ya Ru Yan, Yong Jie Ding, Ning Li, Jian Ping Zhou, Xian Wen Sun, Qing Yun Li

**Affiliations:** 1 Department of Respiratory and Critical Care Medicine, Ruijin Hospital, Shanghai Jiao Tong University School of Medicine, Shanghai, 200025 China; 2 College of Health Science and Technology, Shanghai Jiao Tong University School of Medicine, Shanghai, 200025 China; 3 Institute of Respiratory Diseases, Shanghai Jiao Tong University School of Medicine, Shanghai, 200025 China

**Keywords:** Obesity, Body composition, Respiratory diseases, Mendelian randomization

## Abstract

**Background:**

There is limited evidence on the causal associations of life-course adiposity with the risk of respiratory diseases. This study aimed to elucidate these associations.

**Methods:**

Two-sample Mendelian randomization was conducted using genetic instruments of life-course adiposity (including birth weight, childhood BMI, and adulthood adiposity) to estimate their causal effect on respiratory diseases in participants of European ancestry from the UK Biobank, the FinnGen consortium, and other large consortia.

**Results:**

Genetically predicted higher birth weight was associated with decreased risk of acute upper respiratory infections and increased risk of pulmonary embolism, sleep apnea, and lung cancer. Genetically predicted high childhood BMI was associated with increased risk of asthma, COPD, pulmonary embolism, and sleep apnea. However, most of these observed associations were no longer significant after adjusting for adult BMI. Genetically predicted higher adult BMI and WHR were associated with 10 and 4 respiratory diseases, respectively. High adult body fat percentage and visceral adiposity were genetically associated with increased risk of 9 and 11 respiratory diseases, respectively. Consistently, genetically predicted higher whole-body fat mass was associated with increased risk of 8 respiratory diseases.

**Conclusions:**

This study provides genetic evidence that greater adiposity in childhood and adulthood has a causal effect in increasing the risk of a wide range of respiratory diseases. Furthermore, the effects of childhood obesity on respiratory outcomes may be mediated by adult obesity.

**Supplementary Information:**

The online version contains supplementary material available at 10.1186/s12986-025-00915-2.

## Background

The global prevalence of overweight and obesity continues to rise in recent decades, leading to increasing healthcare costs and the reduction of disease-free years of life [1–3]. Obesity is widely recognized as a significant risk factor of chronic diseases, such as type 2 diabetes, cardiovascular diseases and cancer [4]. Additionally, respiratory diseases are also global health issues that cause substantial morbidity and mortality worldwide [5, 6]. Previous cross-sectional and cohort studies have associated obesity with increased risk of various respiratory diseases, such as obstructive sleep apnea [7], asthma [8], and pulmonary hypertension [9]. However, only a handful of studies have explored the relationship between obesity and other respiratory diseases. Furthermore, the obesity paradox is observed in for chronic obstructive pulmonary disease (COPD), pulmonary embolism, and infection [10]. Specifically, in the context of COPD, general obesity has been associated with decreased risk of airflow limitation, whereas central obesity increased the risk of airflow limitation [11].Given the limited and inconsistent evidence from observational studies, Mendelian randomization (MR) provides an alternative approach to estimating the causal association between obesity and respiratory diseases by using genetic variants as instrumental variables [12]. Because genetic variants are randomly assorted at conception and cannot be modified by the onset of disease or confounding, MR studies are less affected by reverse causality and confounding [13], to which conventional observational studies are susceptible [14].

Overweight and obesity are commonly assessed using body mass index (BMI), waist-hip ratio (WHR), and body composition. BMI and WHR indicate overall and central obesity, respectively. Body composition is defined as the quantification of fat mass and fat-free mass, as well as the proportions of whole-body mass consisting of fat mass and fat-free mass, such as body fat percentage (BF%), visceral adipose tissue (VAT) mass, whole-body fat mass (WBFM), and whole-body fat-free mass (WBFFM) [15]. Previous MR studies have reported that higher BMI in adulthood increases the risk of respiratory diseases such as infections [16], asthma [17, 18], pulmonary hypertension [19], and lung cancer [20], but it remains largely unclear whether birth weight, childhood BMI, and body composition have causal effects on respiratory diseases.

In this study, we applied MR analysis to assess the causal effect of life-course adiposity, including birth weight, childhood BMI, and adult BMI, WHR, BF%, VAT mass, WBFM, and WBFFM on the risk of 12 respiratory diseases.

## Methods

### Study design

**Fig. 1:**
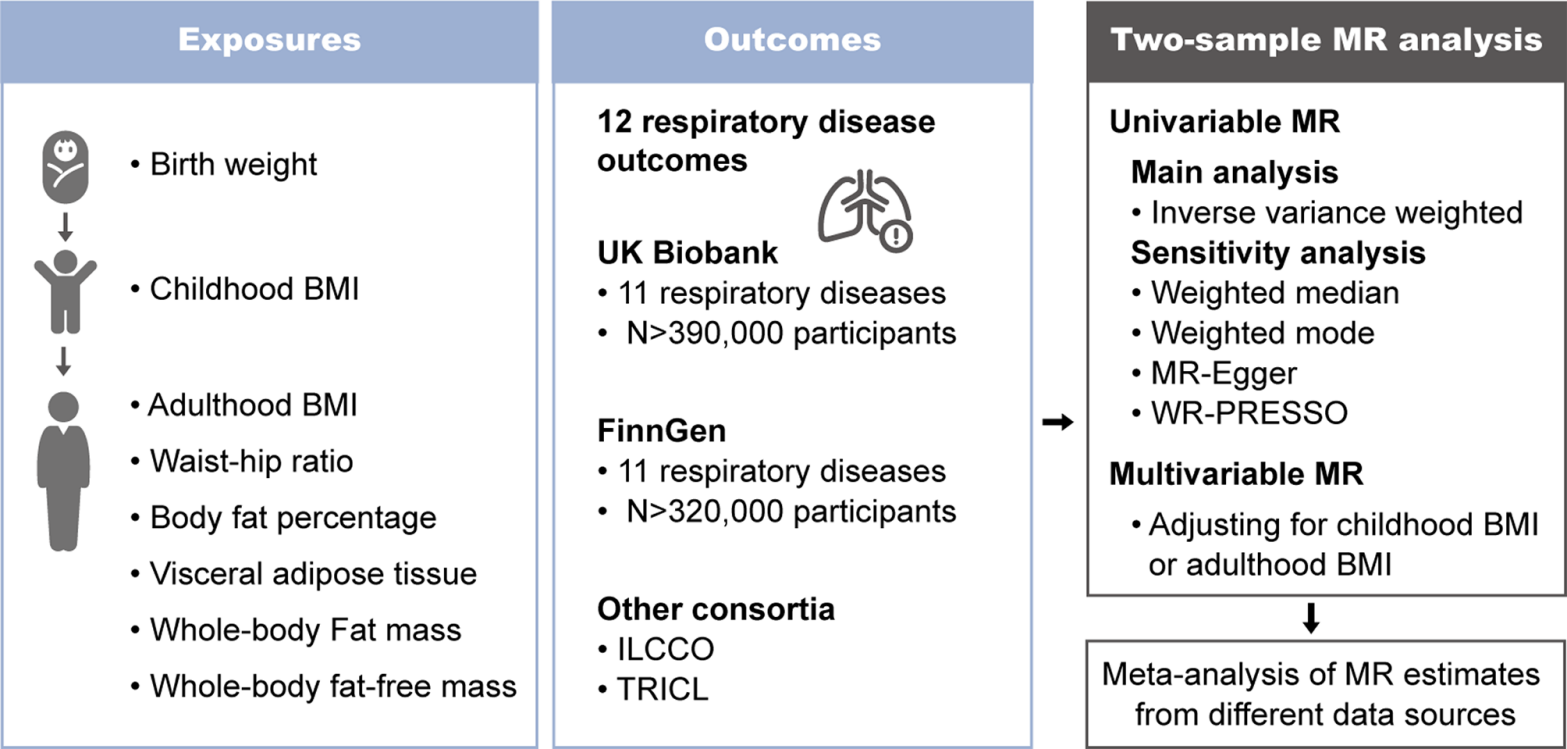
Overview of the MR study design. BMI, body mass index; MR, Mendelian randomization; MR-PRESSO, Mendelian randomization pleiotropy residual sum and outlier; ILCCO, the International Lung Cancer Consortium; TRICL, Transdisciplinary Research Into Cancer of the Lung

This MR study was designed to assess the casual associations of life-course adiposity and body composition with common respiratory diseases, as detailed in Fig. 1. All MR analyses adhere to three fundamental assumptions: (1) genetic variants must be reliably associated with the exposure of interest; (2) genetic variants cannot be associated with confounders affecting the associations between exposures and outcomes; (3) genetic variants should only influence the outcome through the exposure [13]. This study was conducted using publicly available summary-level GWAS data that were obtained from the UK Biobank study, the FinnGen consortium, and large consortia in individuals of European descent (Supplementary Table S1). The ethical approval of each original GWAS has been described in previous studies cited below.

### Data source of exposures

Summary-level statistics for birth weight (*n* = 298,142) and childhood BMI (*n* = 39,620) were obtained from the Early Growth Genetics Consortium (EEG) [21, 22]. Birth weight was collected from measurements at birth, medical records, parental or self-reports [21], and childhood BMI was measured in children between the ages of 2 and 10 years [22]. Summary-level GWAS data for BMI and waist-to-hip ratio (WHR) in adults were from a meta-analysis of up to 806,834 participants of European ancestry [23]. GWAS summary statistics for body composition phenotypes in adults included body fat percentage (BF%, *n* = 454,633) [24], visceral adipose tissue (VAT) mass (*n* = 325,153) [25], whole body fat mass (WBFM, *n* = 454,137) and whole body fat-free mass (WBFFM, *n* = 454,850) [24]. BF%, WBFM and WBFFM were measured using the bioelectrical impedance-based method with the Tanita BC-418MA [24]. VAT mass was estimated by non-linear machine learning models using a large training set of VAT tissue volumes measured by dual-energy X-ray absorptiometry [25]. We also used summary-level statistics obtained from the GWAS of adult BMI (*n* = 322,154) [26] and WHR (*n* = 212,244) [27] from the Genetic Investigation of Anthropometric Traits (GIANT) consortium, and of BF% from a meta-analysis (*n* = 65,831) [28] that did not include GWAS data from the UK Biobank, to minimize sample overlap between the GWAS of adult BMI, WHR, BF%, and outcome data from the UK Biobank in the analysis. We extracted genome-wide significant variants (*P* < 5.00 × 10^− 8^) from the above GWAS data and then used linkage disequilibrium (LD) clumping (r2 < 0.001 within 10,000 kb) based on the 1000 Genomes reference panel to select independent SNPs as genetic instruments for exposures including birth weight, childhood BMI, adult BMI, WHR, BF%, VAT. We then identified and excluded the variants associated with potential confounders such as smoking initiation and daily cigarette consumption using the ‘LDtrait’ function of the ‘LDlinkR’ R package [29], excluded SNPs and detailed genetic instruments are listed in Supplementary Table S2 and S3.

### Data source of outcomes

We included 12 respiratory outcomes to examine the casual effects of adiposity traits in early and later life on common respiratory diseases. Summary-level GWAS data for the 12 respiratory diseases were obtained from the UK Biobank, the FinnGen consortium, the International Lung Cancer Consortium (ILCCO) [30] and the Transdisciplinary Research Into Cancer of the Lung (TRICL) consortium. The UK Biobank is a large-scale prospective cohort study consisting of approximately half a million participants aged 40 to 69 years recruited from across the UK between 2006 and 2010. The FinnGen study is a large genetic research project collecting and analyzing the genome and digital health register data of up to 500,000 Finnish biobank participants [31]. The summary-level statistics for respiratory diseases in individuals of European ancestry used in this study were extracted from the GWAS performed by the Pan-UKB team on UK Biobank data (https://pan.ukbb.broadinstitute.org) and from the FinnGen R10 GWAS results. In addition, GWAS summary statistics for lung cancer were obtained from the ILCCO (11,348 cases and 15,861 controls) and the TRICL consortium (11,245 cases and 54,619 controls) [30], which include individuals of European ancestry. The detailed definitions of respiratory diseases using ICD codes are presented in Supplementary Table S4.

### Statistical analysis

Two-sample univariable MR was performed to assess the causal associations of each exposure (including birth weight, childhood obesity, adulthood BMI, WHR, BF%, and VAT) with 12 respiratory diseases. The inverse variance weighted (IVW) method was applied as the main analysis in univariable MR, and other complementary MR methods, including weighted median, weighted mode, MR-Egger, and MR PRESSO were applied to validate the robustness of the IVW estimates under different MR assumptions. The weighted median method provides consistent causal estimates when up to 50% of the total weight is derived from invalid instrumental variants [32]. The weighted mode method gives an unbiased estimate causal estimates, assuming that the largest number of genetic instruments with similar causal effects are valid [33]. The MR-Egger method can detect the presence of directional pleiotropy (P for MR-Egger intercept < 0.05) and provides conservative effect estimates [34]. The MR-PRESSO method has also been used to assess the presence of directional pleiotropy and to obtain relatively unbiased causal estimates by identifying and excluding potential pleiotropic outliers that cause bias in the MR tests [35]. We assessed the instrument strength by calculating the F statistic and the heterogeneity of the IVW estimates by Cochran’s Q test.

Considering that adiposity in later life is a potential confounder for the observed associations between genetically predicted birth weight and respiratory diseases, we performed multivariable MR analysis to estimate the direct effect of birth weight on each identified respiratory disease after adjusting for childhood BMI or adult BMI. Similarly, we performed multivariable MR analysis to investigate whether genetically predicted childhood BMI had direct effects on the identified respiratory diseases after adjusting for adult BMI. We also performed multivariable MR analysis to estimate the direct effect of genetically predicted WHR, BF%, and VAT volume on respiratory outcomes after adjusting for adult BMI. In addition, multivariable MR was used to estimate the causal effect of genetically predicted WBFM and WBFFM, two highly correlated traits, independently on respiratory diseases.

The results of univariable MR and multivariable MR for each respiratory disease from UK Biobank, FinnGen, ILCCO, and TRICL were pooled using fixed-effects meta-analysis. The Benjamini-Hochberg correction was applied to correct for multiple testing. Associations with Benjamini-Hochberg corrected *P* < 0.05 were considered significant, whereas associations with *P* < 0.05 but not passing the Benjamini-Hochberg correction were considered suggestive, suggesting that further studies are needed to confirm these findings. All MR analyses were performed using the ‘TwoSampleMR’, ‘MendelianRandomization’, ‘MVMR’ and ‘metafor’ packages in the R software (version 4.2.3).

## Results

We performed MR analysis to investigate the causal associations of genetically predicted life-course obesity (including birth weight, childhood BMI, adult BMI and WHR), and body composition (including BF%, VAT mass, WBFM and WBFFM) with 12 respiratory diseases. The F statistic of the genetic instruments for each exposure was greater than 10, suggesting sufficient instrument strength (Supplementary Table S5).

### Effect of birth weight on respiratory diseases

**Fig. 2:**
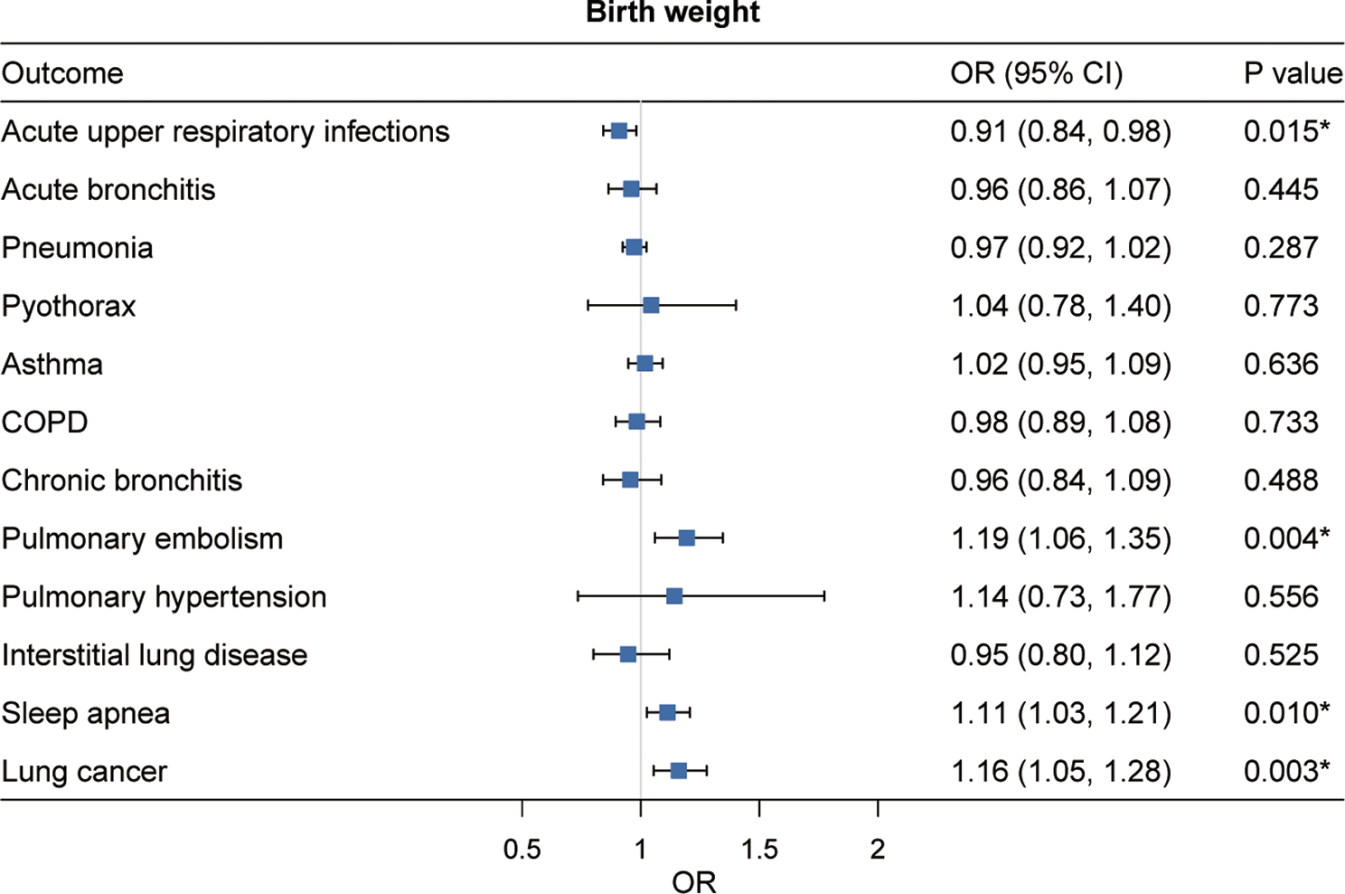
Univariable MR estimates for the causal associations of genetically predicted birth weight with 12 respiratory diseases. OR (95% CI) represents the risk of respiratory diseases associated with each one-SD higher birthweight. *Significant association after multiple testing. COPD, chronic obstructive pulmonary disease; OR, odds ratio; CI, confidence interval

Univariable MR analysis provided evidence of associations between birth weight and several respiratory diseases. In the meta-analysis of IVW estimates from UK Biobank and FinnGen, each genetically predicted one-SD higher birth weight was associated with decreased risk of acute upper respiratory infections (OR: 0.91; 95% CI: 0.84–0.98) and increased risk of pulmonary embolism (1.19; 1.06–1.35), sleep apnea (1.11; 1.03–1.21) and lung cancer (1.16; 1.05–1.28) (Fig. 2). After correction for multiple testing, the association of birth weight with acute upper respiratory infections, pulmonary embolism, sleep apnea and lung cancer remained significant (Fig. 2 and Supplementary Table S6). There was heterogeneity between instrument variables in the analysis of pulmonary embolism and lung cancer, but no horizontal pleiotropy was detected by the MR-Egger intercept analysis (Supplementary Table S7). The genetic association between higher birth weight and decreased risk of acute upper respiratory infections remained significant after adjusting for childhood BMI, or adult BMI. After adjustment for childhood BMI, the positive association of birth weight with lung cancer remained, but the association with pulmonary embolism did not. After adjustment for adult BMI, birth weight remained still positively associated with pulmonary embolism but was no longer significantly associated with lung cancer (Supplementary Table S8).

### Effect of childhood BMI on respiratory diseases

**Fig. 3:**
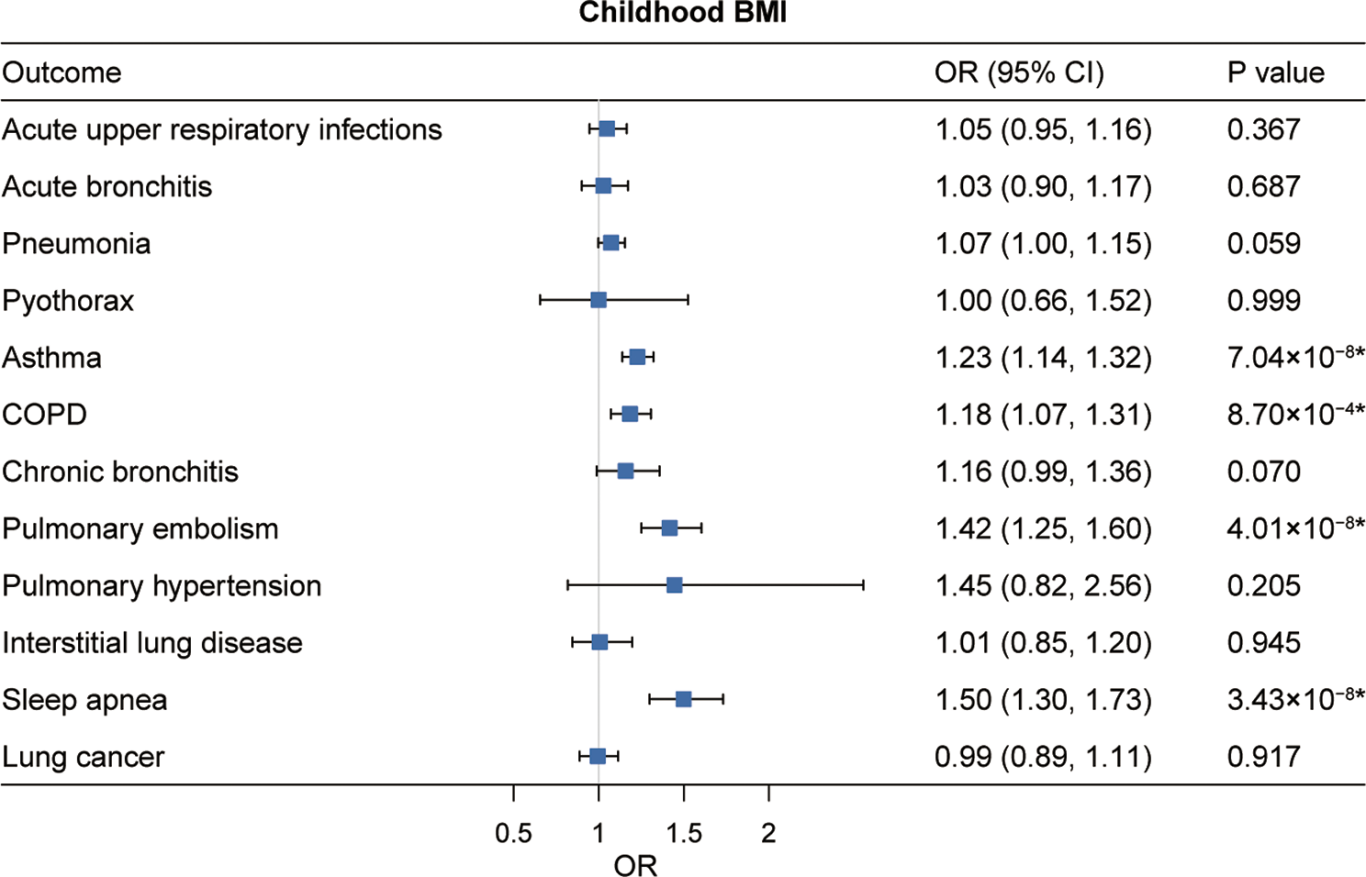
Univariable MR estimates for the causal associations of genetically predicted childhood BMI with 12 respiratory diseases. OR (95% CI) represents the risk of respiratory diseases associated with each one-SD higher childhood BMI. *Significant association after multiple testing. BMI, body mass index; COPD, chronic obstructive pulmonary disease; OR, odds ratio; CI, confidence interval

In the univariable MR analysis, meta-analysis of IVW results showed that each genetically instrumented one-SD higher childhood BMI increased the risk of asthma (OR: 1.23; 95% CI: 1.14–1.32), COPD (1.18; 1.07–1.31), chronic bronchitis (1.17; 1.01–1.35), pulmonary embolism (1.42; 1.25–1.60), and sleep apnea (1.50; 1.30–1.73) (Fig. 3). All these observed associations remained statistically significant after adjustment for multiple comparisons, and these associations were directionally consistent and statistically significant in at least one sensitivity analysis (Fig. 3, Tables S6 and S9). Heterogeneity in instrument effect was observed in the analysis of asthma, COPD, and sleep apnea in the UK Biobank, but no directional pleiotropy was detected (Supplementary Table S9). After adjusting for adult BMI in multivariable MR analysis, genetically predicted childhood BMI was no longer significantly associated with the identified respiratory diseases (Supplementary Table S10).

### Effect of adult BMI and WHR on respiratory diseases

**Fig. 4:**
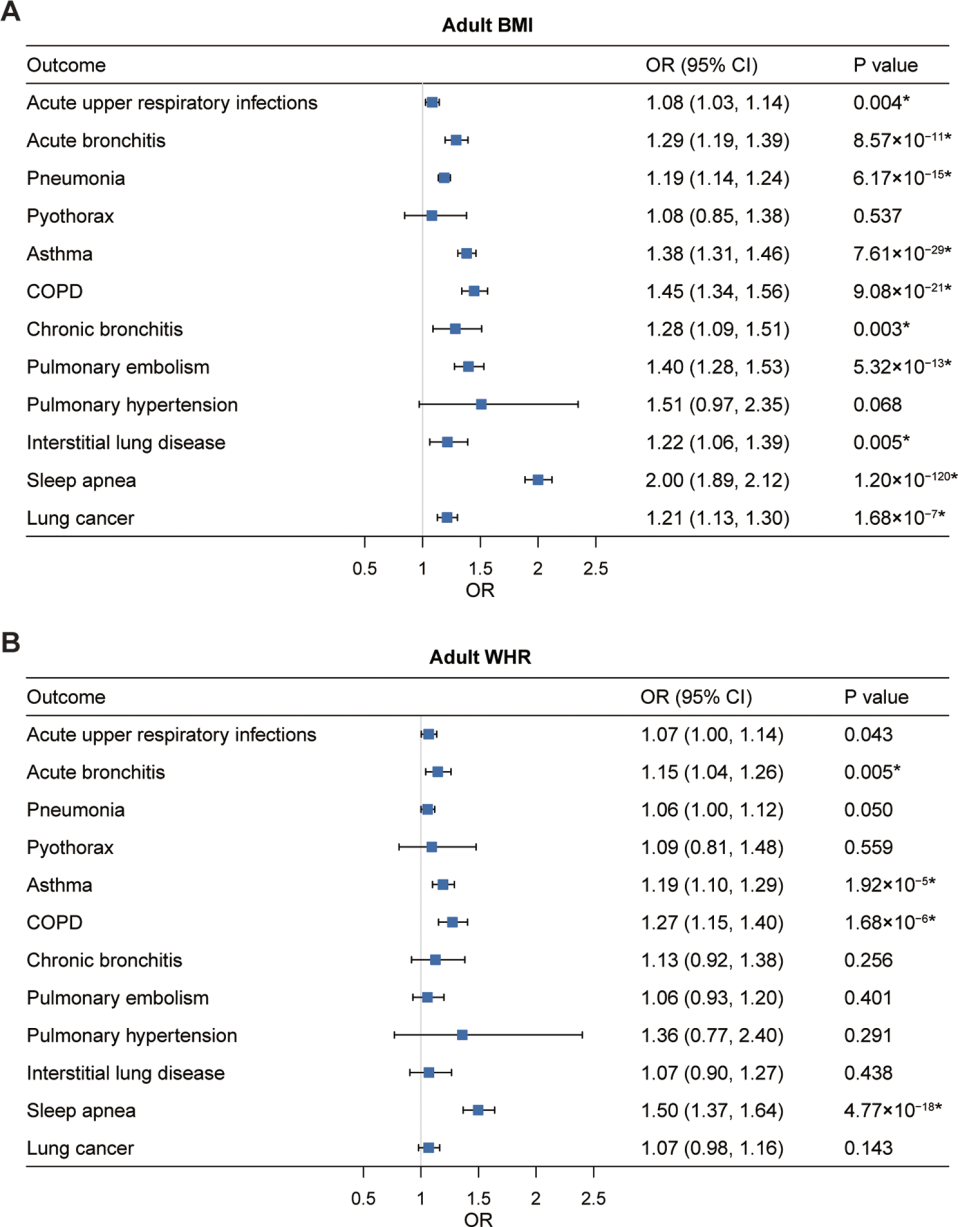
Univariable MR estimates for the causal associations of genetically predicted adult **A**) BMI and **B**) WHR with 12 respiratory diseases. OR (95% CI) represents the risk of respiratory diseases associated with each 1-SD higher adult BMI or WHR. *Significant association after multiple testing. BMI, body mass index; WHR, waist-hip ratio; COPD, chronic obstructive pulmonary disease; OR, odds ratio; CI, confidence interval

The meta-analysis of IVW results from UK Biobank and FinnGen provided evidence that each one-SD increase in genetically instrumented adult BMI was associated with increased risk of 10 of the 12 respiratory diseases, including acute upper respiratory infections (1.08; 1.03–1.14), acute bronchitis (1.29; 1.19–1.39), pneumonia (1.19; 1.14–1.24), asthma (1.38; 1.31–1.46), COPD (1.45; 1.34–1.56), chronic bronchitis (1.28; 1.09–1.51), pulmonary embolism (1.40; 1.28–1.53), interstitial lung disease (1.22; 1.06–1.39), sleep apnea (2.00; 1.89–2.12), and lung cancer (1.21; 1.13–1.30) (Fig. 4A). All these observed associations remained statistically significant after adjustment for multiple comparisons (Fig. 4A and Supplementary Table S6). For the 10 respiratory diseases associated with adult BMI, at least one sensitivity analysis confirmed the associations for outcomes except for acute upper respiratory infections, and interstitial lung disease. MR-PRESSO identified outlying variants for the outcomes, and showed consistent estimates after excluding these outliers (Supplementary Table S11). There was heterogeneity among instrument variables, but no horizontal pleiotropy was found in the MR-Egger intercept analysis (Supplementary Table S11).

Genetically predicted adult WHR had a similar positive effect on the risk of some respiratory diseases. Each one-SD higher genetically predicted WHR increased the risk of acute upper respiratory infections (1.07; 1.00-1.14), acute bronchitis (1.15; 1.04–1.26), pneumonia (1.06; 1.00-1.12), asthma (1.19; 1.10–1.29), COPD (1.27; 1.15–1.40), and sleep apnea (1.50; 1.37–1.64) (Fig. 4B). After correction for multiple testing, the association of adult WHR with acute bronchitis, asthma, COPD, and sleep apnea remained statistically significant, except for the association with lung cancer (Fig. 4B and Supplementary Table S6). The MR-Egger intercept analysis indicated the presence of horizontal pleiotropy for four WHR-disease associations in the FinnGen study, including acute upper respiratory infection, acute bronchitis, pneumonia, and COPD. However, these associations remained significant and consistent in direction after excluding the potentially pleiotropic outliers by MR-PRESSO (Supplementary Table S12). After adjusting for adult BMI in the multivariable MR analysis, the associations of WHR with these respiratory diseases were no longer significant (Supplementary Table S12).

### Association between adult body composition and respiratory diseases

**Fig. 5:**
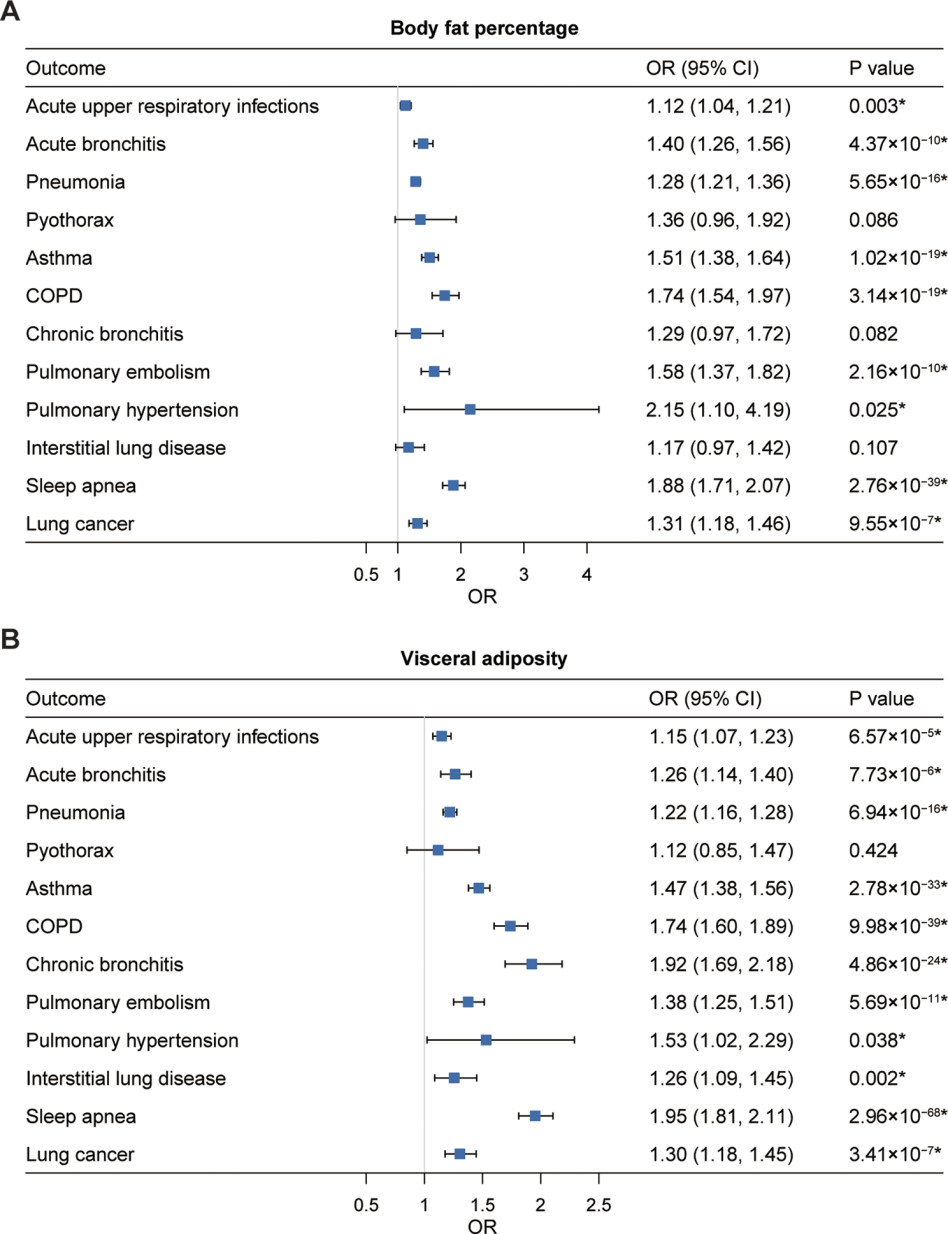
Univariable MR estimates for the causal associations of genetically predicted adult **A**) BF% and **B**) VAT volume with 12 respiratory diseases. OR (95% CI) represents the risk of respiratory diseases associated with each 1-SD higher BF% or VAT volume. *Significant association after multiple testing. BF%, body fat percentage; VAT, visceral adipose tissue; COPD, chronic obstructive pulmonary disease; OR, odds ratio; CI, confidence interval

Meta-analysis of univariable MR results from UK Biobank and FinnGen provided strong evidence that genetically predicted higher BF% was associated with increased risk of 10 of the 12 respiratory diseases investigated, including acute upper respiratory infections (IVW OR: 1.12; 95% CI: 1.04–1.21), acute bronchitis (1.40; 1.26–1.56), pneumonia (1.28; 1.21–1.36), asthma (1.51; 1.38–1.64), COPD (1.74; 1.54–1.97), pulmonary embolism (1.58; 1.37–1.82), pulmonary hypertension (2.15; 1.10–4.19), sleep apnea (1.88; 1.71–2.07), and lung cancer (1.31; 1.18–1.46) (Fig. 5A). All these observed associations remained statistically significant after correction for multiple comparisons (Fig. 5A and Supplementary Table S6). Among the nine respiratory diseases associated with BF%, at least one sensitivity analysis supported the associations for outcomes except for acute upper respiratory infections and pulmonary hypertension (Supplementary Table S13). MR-Egger intercept analysis indicated the presence of directional pleiotropy for acute bronchitis, pulmonary embolism, and sleep apnea. However, these associations remained significant and consistent in direction after excluding the potentially pleiotropic outliers by MR-PRESSO (Supplementary Table S13). After adjusting for adult BMI in multivariable MR analysis, genetically predicted BF% was significantly associated with pneumonia and sleep apnea (Supplementary Table S13).

Genetically determined visceral adipose tissue (VAT) volume had a similar positive effect on 11 of the 12 respiratory diseases studied (Fig. 5B). These VAT-disease associations were still significant after correction for multiple testing (Supplementary Table S6) and remained largely consistent across sensitivity analyses (Supplementary Table S14). MR-Egger pleiotropy test showed no evidence of directional pleiotropy (Supplementary Table S14). After adjusting for the effect of adult BMI, genetically determined VAT mass was still positively associated with chronic bronchitis (Supplementary Table S14).

Multivariable MR analysis evaluating the direct effect of genetically predicted WBFM or WBFFM on the risk of respiratory diseases. In multivariable MR analysis, there were positive associations of genetically predicted WBFM with 8 of 12 respiratory diseases, consistent with the MR results for BF% and VAT (Supplementary Table S15). Increased genetically predicted WBFFM was associated with decreased risk of asthma and COPD, and increased risk of pulmonary embolism after correction for multiple testing (Supplementary Table S15).

## Discussion

**Fig. 6:**
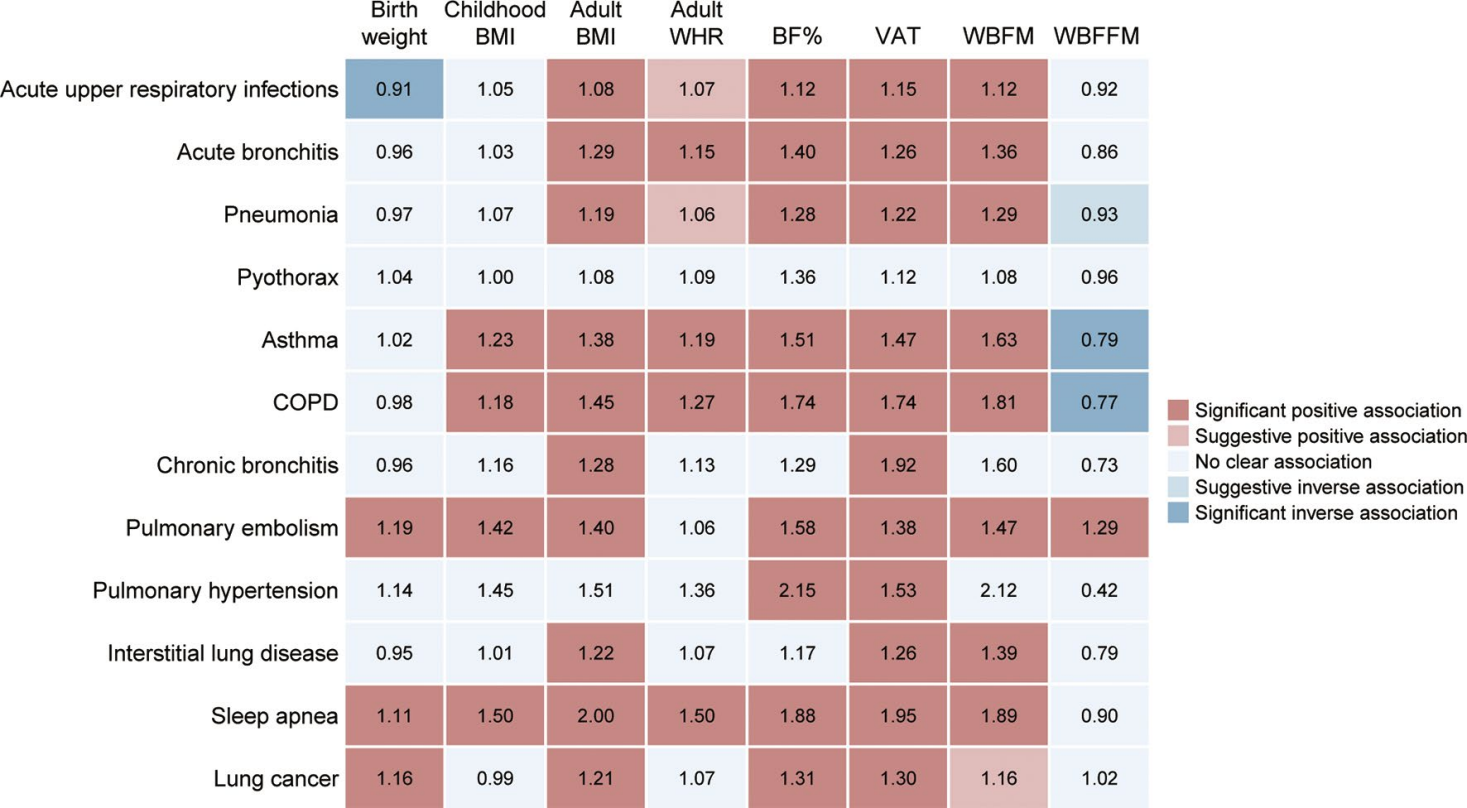
Summary of associations of life-course adiposity and body composition with 12 respiratory diseases. Odds ratios of the associations are indicated by the numbers in the boxes. Associations with Benjamini-Hochberg corrected *P* < 0.05 were considered significant, whereas associations with *P* < 0.05 but not passing the Benjamini-Hochberg correction were considered suggestive. BMI, body mass index; WHR, waist-hip ratio; BF%, body fat percentage; VAT, visceral adipose tissue; WBFM, whole-body fat mass; WBFFM, whole-body fat-free mass; COPD, chronic obstructive pulmonary disease

In this MR study, we examined the causal effect of life-course adiposity (including birth weight, childhood BMI, and adult adiposity) on the risk of 12 respiratory diseases. We showed evidence for the positive associations of genetically determined birth weight and childhood BMI with several respiratory diseases, but the effect of early life adiposity on these outcomes appeared to be mediated by adult adiposity. Our MR analysis also suggested that genetically predicted higher adult BMI, WHR, BF%, visceral adiposity and whole-body fat mass were associated with increased risk of a wide range of respiratory diseases (Fig. 6).

For birth weight, our analysis supported that each genetically predicted one-SD higher birth weight was associated with decreased risk of acute upper respiratory infections, and the protective effect persisted after adjustment for later-life adiposity traits including childhood BMI and adult BMI. We found a significant positive association between genetically predicted birth weight and lung cancer, which is consistent with findings from the Women’s Health Initiative Observational Study that birth weight was positively associated with the risk of several cancers including lung cancer [36]. After adjustment for childhood BMI, we found that the association of birth weight with lung cancer remained. However, after adjustment for adult BMI, birth weight was no longer significantly associated with lung cancer, suggesting that the deleterious effect of high birth weight on lung cancer is mediated by obesity in adulthood. We also provided novel evidence that birth weight was positively associated with pulmonary embolism and sleep apnea, and the genetic association of birth weight with pulmonary embolism remained after adjustment for adult BMI.

Childhood obesity has been identified as a risk factor for asthma in previous observational and MR studies [37, 38], but very few studies have investigated the impact of childhood BMI on the risk of other subsequent adulthood respiratory diseases. Our analysis supported the causal effect of high childhood BMI on asthma, and additionally showed that genetically determined higher childhood BMI was significantly associated with increased risk of COPD, pulmonary embolism, and sleep apnea. However, when adjusted for adult BMI using multivariable MR, the observed associations between high childhood BMI and increased risk of asthma, COPD, pulmonary embolism, and sleep apnea changed toward the null findings. These findings suggest that the effects of childhood obesity on these respiratory outcomes might be mediated by adult obesity.

In adults, epidemiological studies indicate that obesity is a risk factor for the development of several respiratory diseases including asthma, pulmonary embolism, pulmonary hypertension, and pneumonia [39]. A causal effect of high BMI on respiratory diseases from the FinnGen study is supported by a previous MR study [16], and in our analysis these associations were further supported by meta-analysis of MR estimates by using different data sources. We also found evidence for the positive associations between genetically predicted WHR and most respiratory disease outcomes.

Body composition provides more accurate measurements than BMI and WHR. BF% is increasingly recognized as a more accurate predictor of cardiovascular disease risk than BMI or waist circumference [40] due to its ability to distinguish between fat-free mass and fat mass. High BF% is associated with reduced lung function [41], even in individuals with normal weight [42]. We provided novel genetic evidence that high BF% was associated with increased risk of a wide range of respiratory diseases, including acute upper respiratory infections, acute bronchitis, pneumonia, asthma, COPD, pulmonary embolism, pulmonary hypertension, sleep apnea, and lung cancer. In addition, we used multivariable MR to estimate the direct effect of whole-body fat mass on respiratory diseases when accounting for whole-body fat-free mass. Our results supported the role of genetically predicted high whole-body fat mass as a risk factor for a wide range of respiratory diseases, which was consistent with the analysis for BF%.

Visceral fat has proven to be more harmful than other fat depots [25, 43]. Observational studies have positively associated visceral adiposity with obstructive sleep apnea [44] and asthma, and inversely with lung function [45], but studies in other respiratory diseases are limited. Our MR investigation provided causal evidence for the positive association of genetically determined VAT volume with increased risk of all respiratory diseases studied except pyothorax, highlighting the importance of managing visceral adiposity to reduce the risk of respiratory diseases.

The key strength of our investigation was the MR design, which mitigated confounding and reverse causality. Moreover, the valid genetic instrument variables from summary-level GWASs of life-course adiposity and body composition, the large study population from the UK Biobank and FinnGen for respiratory disease outcomes improved the statistical efficiency. Certainly, this study has several limitations. First, although the results of the sensitivity analysis are broadly consistent and indicate limited horizontal pleiotropy, residual horizontal pleiotropy could still bias some results. Second, the MR estimates reflect the effects of genetically determined adiposity at different life stages on respiratory diseases, which might not be directly equivalent to the effects of changes in body weight due to lifestyle modification. Third, we could not estimate non-linear effects between adiposity and respiratory disease outcomes by using summary-level GWAS data. Fourth, our findings were based on GWASs conducted in participants of European ancestry, and therefore the generalization of our findings to other ancestries requires further investigation.

In conclusion, this MR study provides genetic evidence that greater adiposity in childhood and adulthood has a causal effect in increasing the risk of a wide range of respiratory diseases. Furthermore, the effects of childhood obesity on respiratory outcomes may be mediated by adult obesity.

## Electronic supplementary material

Below is the link to the electronic supplementary material.

Supplementary Material 1

## Data Availability

All data sources are listed in Additional file 1: Table S1.

## References

[1] Afshin A, Forouzanfar MH, Reitsma MB, et al. Health effects of overweight and obesity in 195 countries over 25 years. N Engl J Med. 2017;377(1):13–27. 28604169 10.1056/NEJMoa1614362 PMC5477817

[2] Biener A, Cawley J, Meyerhoefer C. The high and rising costs of obesity to the US health care system. J Gen Intern Med. 2017;32(Suppl 1):6–8. 28271429 10.1007/s11606-016-3968-8 PMC5359159

[3] Nyberg ST, Batty GD, Pentti J, et al. Obesity and loss of disease-free years owing to major non-communicable diseases: a multicohort study. Lancet Public Health. 2018;3(10):e490–7. 30177479 10.1016/S2468-2667(18)30139-7 PMC6178874

[4] Guh DP, Zhang W, Bansback N, Amarsi Z, Birmingham CL, Anis AH. The incidence of co-morbidities related to obesity and overweight: a systematic review and meta-analysis. BMC Public Health. 2009;9:88. 19320986 10.1186/1471-2458-9-88 PMC2667420

[5] Prevalence and attributable health burden of chronic respiratory diseases. 1990–2017: a systematic analysis for the global burden of disease study 2017. Lancet Respir Med. 2020;8(6):585–96. 32526187 10.1016/S2213-2600(20)30105-3 PMC7284317

[6] Global burden of chronic respiratory diseases and risk factors. 1990–2019: an update from the global burden of disease study 2019. EClinicalMedicine. 2023;59:101936. 37229504 10.1016/j.eclinm.2023.101936 PMC7614570

[7] Senaratna CV, Perret JL, Lodge CJ, et al. Prevalence of obstructive sleep apnea in the general population: A systematic review. Sleep Med Rev. 2017;34:70–81. 27568340 10.1016/j.smrv.2016.07.002

[8] Beuther DA, Weiss ST, Sutherland ER. Obesity and asthma. Am J Respir Crit Care Med. 2006;174(2):112–9. 16627866 10.1164/rccm.200602-231PP PMC2662903

[9] Frank RC, Min J, Abdelghany M, et al. Obesity is associated with pulmonary hypertension and modifies outcomes. J Am Heart Assoc. 2020;9(5):e014195. 32079475 10.1161/JAHA.119.014195 PMC7335575

[10] Lavie CJ, Sanchis-Gomar F, Neeland IJ. Body composition and pulmonary diseases. Copd. 2022;19(1):262–4. 35604833 10.1080/15412555.2022.2070465

[11] Zhang X, Chen H, Gu K, Jiang X. Association of body mass index and abdominal obesity with the risk of airflow obstruction: National health and nutrition examination survey (NHANES) 2007–2012. Copd. 2022;19(1):99–108. 35385365 10.1080/15412555.2022.2032627

[12] Lawlor DA, Harbord RM, Sterne JA, Timpson N, Davey Smith G. Mendelian randomization: using genes as instruments for making causal inferences in epidemiology. Stat Med. 2008;27(8):1133–63. 17886233 10.1002/sim.3034

[13] Davies NM, Holmes MV, Davey Smith G. Reading Mendelian randomisation studies: a guide, glossary, and checklist for clinicians. BMJ. 2018;362:k601. 30002074 10.1136/bmj.k601 PMC6041728

[14] Sekula P, Del Greco MF, Pattaro C, Köttgen A. Mendelian randomization as an approach to assess causality using observational data. J Am Soc Nephrol. 2016;27(11):3253–65. 27486138 10.1681/ASN.2016010098 PMC5084898

[15] Holmes CJ, Racette SB. The utility of body composition assessment in nutrition and clinical practice: an overview of current methodology. Nutrients. 2021;13(8). 10.3390/nu13082493 PMC8399582 34444653

[16] Yang W, Yang Y, Guo Y, Guo J, Ma M, Han B. Obesity and risk for respiratory diseases: a Mendelian randomization study. Front Endocrinol. 2023;14. 10.3389/fendo.2023.1197730 PMC10497775 37711902

[17] Liu J, Xu H, Cupples LA, O’ Connor GT, Liu C-T. The impact of obesity on lung function measurements and respiratory disease: A Mendelian randomization study. Ann Hum Genet. 2023;87(4):174–83. 37009668 10.1111/ahg.12506 PMC10293090

[18] Sun Y-Q, Brumpton BM, Langhammer A, Chen Y, Kvaloy K, Mai X-M. Adiposity and asthma in adults: a bidirectional Mendelian randomisation analysis of the HUNT study. Thorax. 2020;75(3):202–8. 31611343 10.1136/thoraxjnl-2019-213678

[19] Thayer TE, Levinson RT, Huang S, et al. BMI is causally associated with pulmonary artery pressure but not hemodynamic evidence of pulmonary vascular remodeling. Chest. 2021;159(1):302–10. 32712226 10.1016/j.chest.2020.07.038 PMC8008481

[20] Carreras-Torres R, Johansson M, Haycock PC et al. Obesity, metabolic factors and risk of different histological types of lung cancer: A Mendelian randomization study. PLoS ONE. 2017;12(6). 10.1371/journal.pone.0177875 PMC5464539 28594918

[21] Warrington NM, Beaumont RN, Horikoshi M, et al. Maternal and fetal genetic effects on birth weight and their relevance to cardio-metabolic risk factors. Nat Genet. 2019;51(5):804–14. 31043758 10.1038/s41588-019-0403-1 PMC6522365

[22] Vogelezang S, Bradfield JP, Ahluwalia TS, et al. Novel loci for childhood body mass index and shared heritability with adult cardiometabolic traits. PLoS Genet. 2020;16(10):e1008718. 33045005 10.1371/journal.pgen.1008718 PMC7581004

[23] Yengo L, Sidorenko J, Kemper KE, et al. Meta-analysis of genome-wide association studies for height and body mass index in ∼700000 individuals of European ancestry. Hum Mol Genet. 2018;27(20):3641–9. 30124842 10.1093/hmg/ddy271 PMC6488973

[24] Hemani G, Zheng J, Elsworth B et al. The MR-Base platform supports systematic causal inference across the human phenome. Elife. 2018;7. 10.7554/eLife.34408 PMC5976434 29846171

[25] Karlsson T, Rask-Andersen M, Pan G, et al. Contribution of genetics to visceral adiposity and its relation to cardiovascular and metabolic disease. Nat Med. 2019;25(9):1390–5. 31501611 10.1038/s41591-019-0563-7

[26] Locke AE, Kahali B, Berndt SI, et al. Genetic studies of body mass index yield new insights for obesity biology. Nature. 2015;518(7538):197–206. 25673413 10.1038/nature14177 PMC4382211

[27] Shungin D, Winkler TW, Croteau-Chonka DC, et al. New genetic loci link adipose and insulin biology to body fat distribution. Nature. 2015;518(7538):187–96. 25673412 10.1038/nature14132 PMC4338562

[28] Lu Y, Day FR, Gustafsson S, et al. New loci for body fat percentage reveal link between adiposity and cardiometabolic disease risk. Nat Commun. 2016;7:10495. 26833246 10.1038/ncomms10495 PMC4740398

[29] Myers TA, Chanock SJ, Machiela MJ. LDlinkR: an R package for rapidly calculating linkage disequilibrium statistics in diverse populations. Front Genet. 2020;11:157. 32180801 10.3389/fgene.2020.00157 PMC7059597

[30] Wang Y, McKay JD, Rafnar T, et al. Rare variants of large effect in BRCA2 and CHEK2 affect risk of lung cancer. Nat Genet. 2014;46(7):736–41. 24880342 10.1038/ng.3002 PMC4074058

[31] Kurki MI, Karjalainen J, Palta P, et al. FinnGen provides genetic insights from a well-phenotyped isolated population. Nature. 2023;613(7944):508–18. 36653562 10.1038/s41586-022-05473-8 PMC9849126

[32] Bowden J, Davey Smith G, Haycock PC, Burgess S. Consistent Estimation in Mendelian randomization with some invalid instruments using a weighted median estimator. Genet Epidemiol. 2016;40(4):304–14. 27061298 10.1002/gepi.21965 PMC4849733

[33] Hartwig FP, Davey Smith G, Bowden J. Robust inference in summary data Mendelian randomization via the zero modal Pleiotropy assumption. Int J Epidemiol. 2017;46(6):1985–98. 29040600 10.1093/ije/dyx102 PMC5837715

[34] Bowden J, Davey Smith G, Burgess S. Mendelian randomization with invalid instruments: effect Estimation and bias detection through Egger regression. Int J Epidemiol. 2015;44(2):512–25. 26050253 10.1093/ije/dyv080 PMC4469799

[35] Verbanck M, Chen CY, Neale B, Do R. Detection of widespread horizontal Pleiotropy in causal relationships inferred from Mendelian randomization between complex traits and diseases. Nat Genet. 2018;50(5):693–8. 29686387 10.1038/s41588-018-0099-7 PMC6083837

[36] Spracklen CN, Wallace RB, Sealy-Jefferson S, et al. Birth weight and subsequent risk of cancer. Cancer Epidemiol. 2014;38(5):538–43. 25096278 10.1016/j.canep.2014.07.004 PMC4188724

[37] Chen Y-C, Kuo H-P, Hsia S-M, Wu H-T, Pan W-H, Lee YL. Life course body mass index through childhood and young adulthood and risks of asthma and pulmonary function impairment. Pediatr Pulmonol. 2021;56(5):849–57. 33270354 10.1002/ppul.25197

[38] Au Yeung SL, Li AM, Schooling CM. A life course approach to elucidate the role of adiposity in asthma risk: evidence from a Mendelian randomisation study. J Epidemiol Commun Health. 2021;75(3):277–81. 10.1136/jech-2020-213745 33051271

[39] Murugan AT, Sharma G. Obesity and respiratory diseases. Chron Respir Dis. 2008;5(4):233–42. 19029235 10.1177/1479972308096978

[40] Byambasukh O, Eisenga MF, Gansevoort RT, Bakker SJ, Corpeleijn E. Body fat estimates from bioelectrical impedance equations in cardiovascular risk assessment: the PREVEND cohort study. Eur J Prev Cardiol. 2019;26(9):905–16. 30791699 10.1177/2047487319833283 PMC6545622

[41] Sutherland TJ, McLachlan CR, Sears MR, Poulton R, Hancox RJ. The relationship between body fat and respiratory function in young adults. Eur Respir J. 2016;48(3):734–47. 27471202 10.1183/13993003.02216-2015

[42] Chen YY, Kao TW, Fang WH, et al. Body fat percentage in relation to lung function in individuals with normal weight obesity. Sci Rep. 2019;9(1):3066. 31217470 10.1038/s41598-019-38804-3 PMC6584631

[43] Fox CS, Massaro JM, Hoffmann U, et al. Abdominal visceral and subcutaneous adipose tissue compartments: association with metabolic risk factors in the Framingham heart study. Circulation. 2007;116(1):39–48. 17576866 10.1161/CIRCULATIONAHA.106.675355

[44] Kritikou I, Basta M, Tappouni R, et al. Sleep Apnoea and visceral adiposity in middle-aged male and female subjects. Eur Respir J. 2013;41(3):601–9. 22743670 10.1183/09031936.00183411 PMC7091531

[45] Wu T, Jahangir MR, Mensink-Bout SM, Klein S, Duijts L, Oei EHG. Visceral adiposity and respiratory outcomes in children and adults: a systematic review. Int J Obes (Lond). 2022;46(6):1083–100. 35190670 10.1038/s41366-022-01091-6

